# Rift Valley Fever, Mayotte, 2007–2008

**DOI:** 10.3201/eid1504.081045

**Published:** 2009-04

**Authors:** Daouda Sissoko, Claude Giry, Philippe Gabrie, Arnaud Tarantola, François Pettinelli, Louis Collet, Eric D’Ortenzio, Philippe Renault, Vincent Pierre

**Affiliations:** Cellule Interrégionale d’Épidémiologie de la Réunion et Mayotte, la Réunion (D. Sissoko, E. D’Ortenzio, P. Renault, V. Pierre); Centre Hospitalier de Mayotte, Mayotte (C. Giry, P. Gabrie, F. Pettinelli, L. Collet); Institut de Veille Sanitaire, Saint Maurice, France (A. Tarantola)

**Keywords:** Zoonoses, vector-borne infections, Rift Valley fever virus, phlebovirus, surveillance, Mayotte, Indian Ocean, dispatch

## Abstract

After the 2006–2007 epidemic wave of Rift Valley fever (RVF) in East Africa and its circulation in the Comoros, laboratory case-finding of RVF was conducted in Mayotte from September 2007 through May 2008. Ten recent human RVF cases were detected, which confirms the indigenous transmission of RFV virus in Mayotte.

Rift Valley fever (RVF) is a zoonotic and mosquito-borne disease that affects animals and humans (*1*). In humans, RVF infection is usually asymptomatic or characterized by an acute self-limited febrile illness. However, in ≈1%–3% of case-patients, it can cause serious manifestations that may lead to death or disabling sequelae in survivors (*2**,**3*). Identified in the 1930s in Kenya, RVF virus (RVFV) circulates now in many other African countries (*4**–**7*), as well as on the Arabian peninsula (*8*), where epizootics and associated human cases have been reported.

In early 2007, RVF outbreaks were reported in several eastern African countries (*9*). Additionally, in August 2007, autochthonous RVF transmission was found on the Indian Ocean island of Grande Comore, Republic of Comoros. A young Comorian with a 2-month history of severe encephalitis was transferred from the Republic of Comoros to Mayotte, where diagnostic and medical care facilities are more readily available. Mayotte, a French territory, is located northwest of Madagascar, in the Comoros archipelago (Figure).

**Figure:**
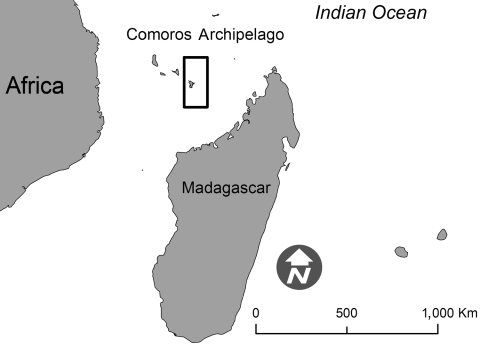
Location of Mayotte (boxed) in the Comoros Archipelago. Source: Préfecture de Mayotte.

Anti-RVFV immunoglobulin (Ig) M was detected by IgM antigen-capture ELISA (*10*) in the serum sample of this patient. This sentinel event had substantial public health implications for Mayotte because the Republic of Comoros and Mayotte have frequent exchanges of populations and goods, both legally and illegally.

An active laboratory-based surveillance system for RVF was implemented for livestock in Mayotte in March 2008, which has led to several recent RVF cases being detected in cattle (Bureau of Veterinary Services, Mayotte, unpub. data). Subsequently, to assess the situation of RFV among humans, we retrospectively and prospectively analyzed serum specimens from febrile patients sampled by a routine surveillance program for chikungunya and dengue viruses. We describe the clinical, biological, and epidemiologic characteristics of human RVF cases in Mayotte.

## The Study

Since the 2005–2006 chikungunya outbreak (*11*), laboratory-based surveillance of chikungunya virus (CHIKV) and dengue virus (DENV) is routinely maintained in Mayotte. Specimens are collected from patients attending local primary healthcare facilities or hospitals who seek treatment for dengue-like illness, i.e., acute onset of high fever accompanied by at least 2 of the following signs: arthralgia, body pain, headache, malaise, fatigue, or rash. Stored patient samples collected from September 2007–March 2008 and prospective specimens from this system, which initially tested negative for CHIKV and DENV as well as for *Plasmodium* spp. by using the rapid diagnostic test OptiMAL (Flow Inc., Portland, OR, USA), were screened for RVFV. We defined a confirmed recent human case-patient as a person with dengue-like illness and the serum positive for anti-RVFV IgM by antigen-capture ELISA or RVFV RNA by reverse transcriptase–PCR (*12*). Any specimen positive when tested by the local reference laboratory was then forwarded for confirmation to the National Reference Centre for Arboviruses and the World Health Organization Collaborating Centre for Arboviruses, Pasteur Institute, Paris, France. Identified case-patients were investigated by using a standardized form to gather information, particularly regarding exposure data, e.g., peridomestic environment, handling of animal products, occupational exposures, and travel history. The relevant period of exposure was 3 weeks before disease onset. Medical records of confirmed case-patients were also reviewed if needed. Investigations were conducted in accordance with the French National Institute of Public Health (Institut de Veille Sanitaire) guidelines for studies conducted in rapid response to public health threats.

From September 1, 2007, through May 31, 2008, 220 serum specimens from persons with dengue-like illness who tested negative for *Plasmodium* spp., CHIKV, and DENV were screened; recent RVFV infection (i.e., presence of viral RNA or IgM) was found in 10 samples (4.5%). The earliest recorded onset of dengue-like illness was September 27, 2007, and the latest was May 14, 2008. Seven cases (70%) occurred from January through April, during the hot, rainy season (Table). Confirmed case-patients were not clustered spatially and resided in the following counties: Mamoudzou (3), Bandraboua (2), Dembeni (1), Sada (1), Chirongui (2), and Boueni (1).

**Table:** Epidemiologic, clinical, and laboratory findings of 10 case-patients with confirmed recent Rift Valley fever virus infection, Mayotte, France, September 2007–May 2008*

Case-patient no.	Age, y/sex, occupation	Identified source of exposure(s)	Onset date; early signs and symptoms	Laboratory findings
1	47/M, gardener	Herding animals	2007 Sep 27; 1-day history of fever, arthralgia, body pain	RT-PCR positive, AST 9N, ALT 4N
2	42/F, shopkeeper	Contact with aborted animal	2008 Jan 5; 2-day history of fever, headache, malaise	RT-PCR positive
3	21/M, unknown	Unknown	2008 Jan 19; chronic hepatitis B, 3-day-history of fever, arthralpgia, and headache	RT-PCR positive, AST 4 N, ALT 3N, elevated CPK level, thrombocytopenia
4	19/M, farmer and herder of animals	Milked animal	2008 Jan 21; 2-day history of fever, joint pain, headache, malaise	RT-PCR positive
5	16/M, student	Killed and skinned sick animals	2008 Jan 21; 2-day history of fever, joint pain, headache	RT-PCR positive
6	44/M, unemployed	Mosquito breeding sites in rural household	2008 Mar 13; 2-day history of fever, body aches; hepatic encephalopathy	RT-PCR positive (threshold values), RVF IgM positive
7	19/M, student	Undetermined	2008 Mar 19; 1-day history of fever, myalgia, retro-orbital pain, joint pain, body aches, malaise	RT-PCR positive
8	24/M, handyman	Numerous mosquito breeding sites in household	2008 Mar 28; 5-day history of fever, joint pain, myalgia, nausea/vomiting	RT-PCR positive at threshold, RVF IgM positive, thrombocyopenia
9	32/M, construction worker	Mosquito breeding sites in rural household	2008 May 5; 7-day history of fever, myalgia, joint and body pain, malaise	RVF IgM positive, elevated ALT, AST, and CPK levels
10	53/M, farmer	Slaughtering and butchering animals	2008 May 14; 5-day history of fever, joint pain, body aches and symptoms of right-sided heart failure, discharge diagnosis of pericarditis; new episode of fever, fatigue, and shortness of breath 30 days later and screening for RVF	RVF IgM positive

*RT-PCR; reverse transcription–PCR; AST, aspartate aminotransferase; N, upper limit of normal titers; ALT, alanine aminotransferase; CPK, creatine phosphokinase; RVF, Rift Valley fever; Ig, immunoglobulin.

Of these confirmed-case patients, 9 (90%) were male (age range 16–53 years, median 27.5 years), 5 (50%) were French citizens native to Mayotte, and 5 (50%) were Comorian citizens. None of the cases reported recent (i.e., within 3 weeks before onset of illness) travel into countries with RVF endemic transmission.

Although 2 patients were admitted to the intensive care unit for medical management, no severe neurologic manifestations of RVF were observed, and no deaths could be attributed to RVF. Case-patient 6 (Table), with a coexisting condition of cirrhosis after hepatitis B infection, had substantial thrombopenia (< 40,000 cells/mm^3^) and died as a result of gastrointestinal bleeding and hepatic encephalopathy. Case-patient 10 was admitted to the hospital in May 2008 with a 5-day history of high body temperature, body pain, joint pain (particularly in the shoulders), and fatigue, combined with symptoms consistent with right-sided heart failure (shortness of breath, hepatomegaly, and peripheral edema). The patient was discharged with a diagnosis of pericarditis. He had a follow-up visit 1 month later, and a sample obtained on this occasion tested positive for RVFV IgM and IgG.

Predictably, contact with ruminants (sheep, cattle, or goats) was the predominant means of exposure among reported case-patients; this mode was documented in 5 of 9 cases that were fully investigated. Furthermore, only the presence of numerous breeding sites for mosquitoes in the housing environment remained as a possible means of exposure for 3 other case-patients. The mode of transmission remains undetermined for the remaining case-patient, who had no admitted direct contact with ruminants and whose housing environment did not have numerous breeding sites (Table).

## Conclusions

The laboratory case-finding initiated in response to the 2006–2007 RVFV outbreaks in eastern Africa found indigenous transmission in Mayotte. As a result, information was provided to the population, and preventive activities were undertaken, e.g., information for healthcare providers, a campaign to eliminate mosquito-breeding sites, recommendations to avoid contact with mosquitoes and sick animals, and instructions on adopting appropriate protective measures when engaging in activities related to animals, including slaughtering and butchering.

Because our investigations were mostly retrospective, we cannot exclude recall bias about exposure and incomplete documentation. Moreover, entomologic investigations in the living areas of the case-patients several months after the first signs of illness occurrence were incomplete.

RVF activity in Mayotte appears to be an expansion of the eastern Africa outbreak. As in the case of the chikungunya outbreak in 2005–2006 in Mayotte, thought to have started in Lamu, Kenya (*13*), RVFV circulation illustrates once again the risk of introduction or circulation in Mayotte or other Comorian islands of infectious agents involved in outbreaks in neighboring eastern African coastal countries (*14*). This risk is particularly important because of the favorable climatic conditions and the presence in Mayotte of replication-competent vectors of numerous arboviruses.

This epidemic is a further illustration that it is imperative for healthcare providers, epidemiologists, and policy makers in Mayotte to remain alert each time there are outbreaks in nearby countries because the risk of extension to Mayotte is always plausible. Furthermore, the documentation of RVFV circulation in Mayotte should serve as a further impetus toward the development of surveillance programs for nonspecific febrile illnesses, especially in settings where arboviruses are highly prevalent. To achieve this objective, local laboratory capabilities should be enhanced to enable biological diagnosis of a wide array of arboviruses that actively circulate in the eastern African seaboard region. Furthermore, medical education materials and feedback bulletins should regularly provide information about accessing laboratory services while encouraging patients with febrile illness to seek early medical evaluation. Ultimately, maintaining such programs is essential to promptly detecting any renewed introduction or resurgence of RVF and other pathogens, especially during the hot and rainy season.
